# Different Subgroups of Cholinergic Neurons in the Basal Forebrain Are Distinctly Innervated by the Olfactory Regions and Activated Differentially in Olfactory Memory Retrieval

**DOI:** 10.3389/fncir.2018.00099

**Published:** 2018-11-13

**Authors:** Yingwei Zheng, Shouya Feng, Xutao Zhu, Wentao Jiang, Pengjie Wen, Feiyang Ye, Xiaoping Rao, Sen Jin, Xiaobin He, Fuqiang Xu

**Affiliations:** ^1^University of Chinese Academy of Sciences (UCAS), Beijing, China; ^2^State Key Laboratory of Magnetic Resonance and Atomic and Molecular Physics, Key Laboratory of Magnetic Resonance in Biological Systems, Wuhan Institute of Physics and Mathematics, Chinese Academy of Sciences, Wuhan, China; ^3^College of Life Sciences, Wuhan University, Wuhan, China; ^4^Huazhong University of Science and Technology (HUST)-Suzhou Institute for Brainsmatics, Suzhou, China; ^5^Center for Excellence in Brain Science and Intelligence Technology, Chinese Academy of Sciences, Shanghai, China

**Keywords:** BFCNs, subpopulation, neuronal circuit, olfactory associated memory, virus tracing tools

## Abstract

The mammalian basal forebrain (BF), a heterogenous structure providing the primary cholinergic inputs to cortical and limbic structures, plays a crucial role in various physiological processes such as learning/memory and attention. Despite the involvement of the BF cholinergic neurons (BFCNs) in olfaction related memory has been reported, the underlying neural circuits remain poorly understood. Here, we combined viral trans-synaptic tracing systems and ChAT-cre transgenic mice to systematically reveal the relationship between the olfactory system and the different subsets of BFCNs. The retrograde adeno-associated virus and rabies virus (AAV-RV) tracing showed that different subregional BFCNs received diverse inputs from multiple olfactory cortices. The cholinergic neurons in medial and caudal horizontal diagonal band Broca (HDB), magnocellular preoptic area (MCPO) and ventral substantia innominate (SI; hereafter HMS complex, HMSc) received the inputs from the entire olfactory system such as the olfactory bulb (OB), anterior olfactory nucleus (AON), entorhinal cortex (ENT), basolateral amygdala and especially the piriform cortex (PC) and hippocampus (HIP); while medial septum (MS/DB) and a part of rostral HDB (hereafter MS/DB complex, MS/DBc), predominantly from HIP; and nucleus basalis Meynert (NBM) and dorsal SI (hereafter NBM complex, NBMc), mainly from the central amygdala. The anterograde vesicular stomatitis virus (VSV) tracing further validated that the major target of the OB to the BF is HMSc. To correlate these structural relations between the BFCNs and olfactory functions, the neurons activated in the BF during olfaction related task were mapped with c-fos immunostaining. It was found that some of the BFCNs were activated in go/no-go olfactory discrimination task, but with different activated patterns. Interestingly, the BFCNs in HMSc were more significantly activated than the other subregions. Therefore, our data have demonstrated that among the different subgroups of BFCNs, HMSc is more closely related to the olfactory system, both structurally and functionally. This work provides the evidence for distinct roles of different subsets of BFNCs in olfaction associated memory.

## Introduction

The basal forebrain (BF) is a complicated structure made up of several sub-regions, all containing GABAergic, cholinergic and glutamatergic neurons (Semba, 2000; Zaborszky et al., 2011). The cholinergic neurons in the BF (BFCNs) are large, and usually intermingled with non-cholinergic neurons (Gritti et al., 2006). Tracing studies suggest the subsets of BFCNs selectively project to different brain areas as revealed by conventional tracers (Mesulam et al., 1983; Saper, 1984; Woolf, 1991; Zaborszky et al., 2008, 2011) as well as viral tracing combined with Cre-line transgenic mice (Záborszky et al., 2015; Gielow and Zaborszky, 2017). Based on the soma locations and the innervation regions, BFCNs are classified into several sub-groups: Ch1/Ch2 locate in the medial septum (MS) and vertical diagonal band Broca (VDB), providing the major projection to the hippocampal formation; Ch3 locates in the horizontal DB (HDB), providing the major innervations to the olfactory bulb (OB); and Ch4 locates in the substantia innominate (SI) and nucleus basalis Meynert (NBM), mainly innervating the neocortices and amygdala (Mesulam et al., 1983; Woolf, 1991). Generally, a given cholinergic neuron has lengthy axon with enormous branches, innervating multiple brain areas (Wu et al., 2014; Kondo and Zaborszky, 2016; Li et al., 2018); and a given brain area can receive inputs from multiple subpopulations of BFCNs, although not equally (Kim et al., 2016; Gielow and Zaborszky, 2017). Despite the structural studies about the olfaction system and BFCNs (Do et al., 2016; Hu et al., 2016), the relations between the olfactory system and the BFCN subsets are still unclear.

Functionally, the BFCNs have been implicated in several physiological processes, such as attention, learning/memory and arouse (Han et al., 2014; Hangya et al., 2015; Harrison et al., 2016; Ni et al., 2016). Parallel to the anatomical heterogeneity of BFCNs, the functions of different subregional BFCNs are diverse (Vale-Martínez et al., 2002; Martin et al., 2008; Okada et al., 2015). Selective lesions of different BFCN subsets induce different effects in multiple behavioral paradigms (Vale-Martínez et al., 2002; Knox and Berntson, 2006; Gold et al., 2011; Jiang et al., 2016; Knox and Keller, 2016). The BFCNs have been shown to be involved in various olfaction related functions (Vale-Martínez et al., 2002; Wilson et al., 2004; Miasnikov et al., 2006). The BFCNs in HDB can modulate the neural excitability of OB, and improve olfactory discrimination, detection and learning (Ma and Luo, 2012; Devore et al., 2014; Bendahmane et al., 2016). Blocking the muscarinic acetylcholine receptors in piriform cortex (PC) induces generalization of odorants, and impairs odor discrimination and learning (Wilson, 2001). Although those results suggest that BFCNs play roles in the olfactory information processing, learning and memory, the relation between the BFCNs subsets and olfaction related memory is not clear.

Here, by using virus-based circuit tracers combined with ChAT-cre transgenic mice to reveal the detailed relationship between the olfactory system and the BFCNs, we found that among the different subpopulations of the BFCNs, HMS complex (HMSc) had the strongest relationship with the olfactory areas. Furthermore, by using c-fos mapping to reveal the activated pattern of BFCNs in go/no-go odor discrimination task, we found that HMSc was also the most strongly involved subsets in this olfactory behavior. These results showed that subgroups of BFCNs had different input patterns from the olfactory system, and that HMSc had the strongest structural and functional connections with the olfactory system.

## Materials and Methods

### Animals

All procedures were approved by the Animal Care and Use Committee (Wuhan Institute of Physics and Mathematics, the Chinese Academy of Sciences). Experiments were performed on adult male C57BL/6 mice purchased from Hunan SJA Laboratory Animal (Changsha, Hunan, China), and ChAT-Cre mice with C57BL/6 background were from Jackson Laboratory (Bar Harbor, ME, USA). The animals were housed on a 12/12 light/dark cycle with ambient temperature (21 ± 1°C) and humidity (50 ± 5%). The adult ChAT-Cre mice of both sexes (2–3 months old, 20–30 g) and the male C57BL/6 mice (8 weeks old, 20–30 g) were used in the study.

### Behavioral Testing

To confirm the activated region of BFCNs in olfactory associated memory, the go/no-go olfactory discrimination task were used as a model. Two odorants, carvone and citronellol, were used for go/no-go training, and carvone was predetermined as rewarding odor. The C57BL/6 mice deprived of water for 24 h were randomly divided into three groups: the first group, without odor and water (Control group); the second, stimulated with these two odorants, but without training and water reward (ONT group); and the last, stimulated with these two odorants and training on go/no-go olfactory discrimination task (GNG group). For all groups, each animal received more than 0.9 ml of water/day, from reward during the experiment or direct delivery. All the training were done during the light phase at a consistent time every day, and the two odors were presented in a random sequence. The head-fixed mice were trained to learn to discriminate the odors with and without water reward. Up to 100 training trails were performed on each mice every day for five consecutive days. The total time that the mice was kept head-fixed was less than 2 h each day. When the accuracy reached 90%, the mice were ready for a test next day. In the test day, the mice received 10 test trails. The behaviors of the mice were recorded by Spike 2.0 software (CED, Cambridge, UK). After 1.5–2 h, the mice were euthanized for histological studies.

### Virus Information

All the viruses in the study were purchased from Brain VTA (BrainVTA Co., Ltd., Wuhan, China). For the detailed information of the viruses, please see the supplemental materials (Supplementary Table S1).

### Surgery and Viral Injections

All virus injection experiments were performed in Biosafety level 2 (BSL-2) animal experimental platform. Animal were anesthetized with pentobarbital sodium (50 mg/kg, i.p.), and head was fixed in stereotaxic apparatus (Item: 68030, RWD, Shenzhen, China). Exposured the skull, thinned the skull in targeted brain area using a dental drill and removed carefully the skull. Virus injection was performed by a syringe pump (Item: 53311, Quintessential stereotaxic injector, Stoelting, Wood Dale, IL, USA) connected with a diameter of 10–15 μm glass micropipette with a speed at 10 nl/min. After finishing the injection, the glass micropipette was maintained for an extra 10 min and slowly retreated. Then, the incision was stitched, and animals were treated with lincomycin hydrochloride and lidocaine hydrochloride gel for alleviating the inflammation and pain.

To investigate the inputs of subregional BFCNs from different olfactory areas, cell type specific trans-monosynaptic AAV-RV tracing system was utilized. In the present study, the BF were manually delineated into three parts based on the previous studies and the actual physical distance in view of neurotropic viral diffusion (Mesulam et al., 1983; Záborszky et al., 1986; Woolf, 1991): MS/DB complex (MS/DBc) included MS, VDB and a part of rostral HDB; HMSc included medial and caudal HDB, magnocellular preoptic area (MCPO) and ventral SI; and NBM complex (NBMc) included dorsal SI and NBM. The skull over the BF was exposed, and a craniotomy was performed. Then, 100 nl of AAV-Dio-GFP-TVA and AAV-Dio-RVG (1:1 titter, 100 nl in total) were injected into right these subregions, respectively. The injection coordinates were MS/DBc (AP: 1.15 mm, ML: 0.98 mm, DV: −4.5 mm, *θ* = 12°C), HMSc (AP: 0.75 mm, ML: −0.90 mm, DV: −5.3 mm) and NBMc (AP: 0.70 mm, ML: −2.0 mm, DV: −4.20 mm), respectively. After 2 weeks, 100 nl of RV-ENVA-ΔG-Dsred was injected into the same site. Seven days later, the mice were euthanized for histological studies (*n* = 9; 3 subjects/group**)**.

To further confirm the inputs of the BFCN subsets, anterograde transsynaptic virus tracer, vesicular stomatitis virus (VSV), was used (Beier et al., 2011). VSV vectors contained its original glycoprotein (G) genes which endowed VSV anterograde transsynaptic abilities. Moreover, VSV has rapid gene expression and relatively high cytotoxicity to infected host neurons, significantly affect their morphologies. With time and viral dosage, the effect gradually increase. So, we used small dosage of VSV for OB labeling. The surgical procedure was the same as above, except the location. One-hundred nanoliter of VSV mixture including VSV and 0.1% CTB (v/v 4:1) was injected into the right OB. The injection coordinates was (AP: 4.7 mm, ML: 0.75 mm, DV: 0.60 mm). The mice were euthanized for histology at 72 and 96 h after virus injection, respectively (*n* = 8, 4 subjects/group**)**.

### Histology and Immunohistochemistry

Mice were deeply anesthetized with isoflurane and perfused with PBS, and followed by 4% paraformaldehyde (w/v in PBS pH = 7.4). The brain was isolated from the skull and fixed in 4% paraformaldehyde overnight, followed by cryoprotection with a 30% sucrose solution 2–3 day at 4°C, then sectioned coronally with 40 μm thickness using a cryostat (Thermo, CRYOSTAR NX50). For adeno-associated virus and rabies virus (AAV-RV) tracing samples, one out of six consecutive slices was stained with DAPI. For VSV tracing samples, all OB slices and one out of six consecutive slices of the other brain regions were stained with DAPI. For all tracing samples, the slices in the BF were also immunostained with ChAT. In go/no-go olfactory discrimination task, one out of three consecutive slices between 1.10 anterior and −0.94 mm to Bregma was chosen for immunostaining with c-fos and ChAT.

For immunostaining, brain sections were washed with PBS three times and blocked for 1 h with blocking buffer (10% normal serum, 0.3% v/v TritonX-100 in PBS) at 37°C, then incubated with primary antibody [goat anti-ChAT antibody (1:200 dilution, catalog# ab144P, Millipore), rabbit anti-c-fos antibody (1:500 dilution, catalog#2250 s, Millipore)] for 3 days at 4°C, washed three times and incubated with the secondary antibody for 2 h at RT (Alexa Flour 647 donkey anti-goat IgG, goat anti-rabbit CY3, goat anti-rabbit 488, 1:1,000 dilution, Jackson). After three times of washing, the sections were stained with DAPI (1:5,000 Invitrogen).

### Imaging, Cell Counting and Topographic Analysis

Fluorescent images of brain slices were obtained from Leica DM-680 microscope (Leica, Germany) and Olympus VS120 microscope (Olympus, Japan). The boundaries of brain regions near the injection site in BF were delineated by Photoshop based on the Mouse Brain Atlas in Stereotaxic Coordinates (Paxinos and Franklin, 2013). In retrograde tracing, the cholinergic neurons were specifically labeled with GFP by AAV-Dio-TVA-GFP. The TVA, a receptor for avian EnvA envelop glycoprotein expressed by the AAV, can be recognized by RV with EnvA-pseudotyped, G-deleted and dsRed. The cholinergic neurons labeled with both AAV (GFP) and RV (dsRed) were starter cells (yellow). To ensure the accuracy of cell counting, the starter cells near the virus injection site were carefully checked to confirm that their locations were restricted to the given range, especially these nearby brain areas, such as the olfactory tubercle (Tu). The data were excluded from the experiment if there were starter cells outside the given subsets. For following counting, the boundary of brain region was delineated based on the Allen Brain Atlas. Then, RV infected neurons across the whole brain were counted using ImageJ software. To reduce the error caused by the different amount of starter cells in different groups, normalization was done by the amount of RV positive neurons in given brain area divided the whole-brain RV positive neurons. CTB is a sensitive tracer, and low concentration can be used to locate the diffusion range of virus. In VSV experiment, a low concentration of CTB 488 (0.1%) was mixed in VSV virus, and the diffusion range of CTB were believed the area of the VSV virus diffusion in injection site. All stained OB slices were checked the range of the green fluorescent neurons to ascertain that diffusion range of VSV was restricted in OB. Similarly, the data were excluded from the experiment if there were CTB diffusion range outside OB. Then, the brain regions and ChAT positive neurons infected by VSV in BF were counted.

In go/no-go olfactory discrimination task, the total number of c-fos and Chat positive cells, and the co-expression neurons of c-fos and Chat were counted. The corresponding average densities and the percentages in different subsets of BF were calculated.

### Data Processing

SPSS (version 13.0) and Origin 9.0 were used for data analysis (one way ANOVA and *post hoc* test) and statistical graphs, respectively. All data were displayed as mean ± SEM. Spearson’s correlation analysis was used to compare the olfactory inputs to the subpopulations of BFCNs. Statistical significance was set as ***P < 0.001, **P < 0.01, and *P < 0.05.

## Results

### Different Subsets of BFCNs Received Inputs From Different Olfactory Regions

**Figure 1:**
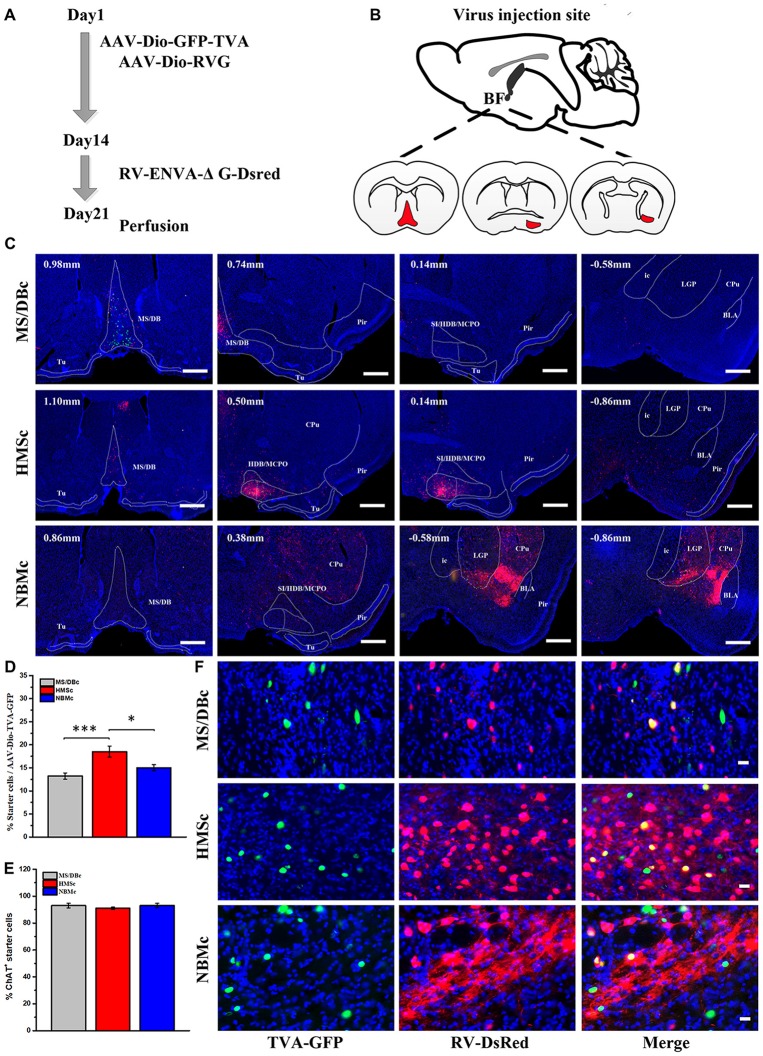
Retrograde AAV-RV viral tracing. **(A)** Time-course of AAV-RV injections for trans-monosynaptic labeling. **(B)** Diagram of RV injection site, medial septum/diagonal band Broca complex (MS/DBc; Left), HMS complex (HMSc; Middle) and nucleus basalis Meynert complex (NBMc; Right). **(C)** Range of starter cells near the injection sites in coronal brain sections, MS/DBc (Upper), HMSc (Middle) and NBMc (Down). The starter cells (yellow) were these co-infected by AAV (green) and RV (red), which were distributed in the given subpopulation range. Scale Bar, 500 μm. **(D)** The percent of starter cells in the neurons infected by AAV-Dio-TVA-GFP. **(E)** The percent of cholinergic starter cells. **(F)** Enlarged views of the starter cells in MS/DBc (Top), HMSc (Middle) and NBMc (Bottom). AAV-Dio-GFP-TVA (Green), RV-ENVA-ΔG-Desred (Red), starter cell (Yellow). Scale bar, 20 μm. *P < 0.05, **P < 0.01 and ***P < 0.001.

**Figure 2:**
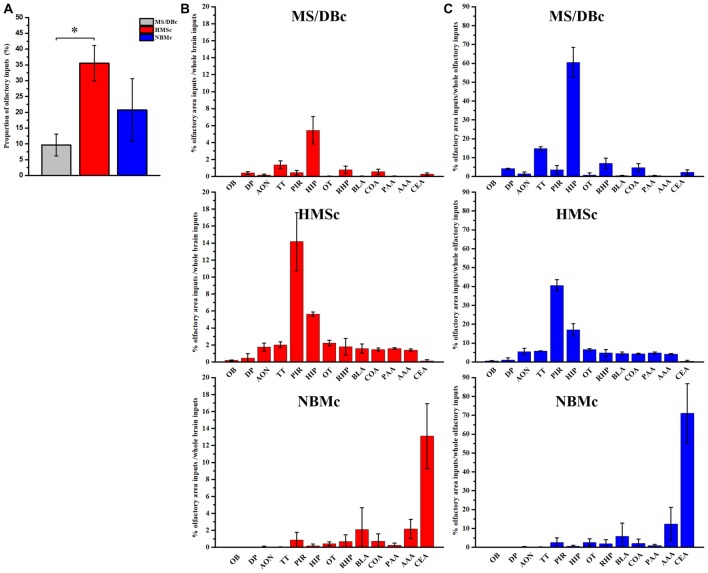
Quantitative analysis of the olfactory inputs onto the different basal forebrain cholinergic neuron (BFCN) subpopulations. **(A)** The percentage of the olfactory inputs onto the subpopulations of BFCNs out of the total inputs across the brain. **(B)** Proportion of every olfactory area inputs and whole brain inputs to cholinergic neurons in the different subpopulations of BFCNs, MS/DBc (Top), HMSc (Middle) and NBMc (Bottom). **(C)** Proportion of every olfactory area inputs and whole olfactory regions to cholinergic neurons in the different subpopulations of BFCNs. MS/DBc (Top), HMSc (Middle) and NBMc (Bottom). *P < 0.05, **P < 0.01 and ***P < 0.001.

AAV-RV based cell-type-specific retrograde tracing system was used to identify the olfactory inputs of BFCN subpopulations (Figures 1A,B for experimental time-course and injection sites, respectively). The cholinergic neurons infected by AAV-Dio-GFP-TVA expressed GFP. Near the injection site, the yellow neurons were the starter cells which were co-infected by AAV-Dio-GFP-TVA and RV-ENVA-ΔG-Dsred (Figure 1F). Since the distribution range of starter cells was found to be located within the assigned BFCN subregions (Figure 1C), the RV positive cells in the other brain regions should be the direct input neurons to the corresponding BFCNs. The numbers of RV positive cells in the whole brain were counted, and the brain areas with a proportion of more than 0.5% had been shown (Supplementary Figure S1). And quantitative input proportion of multiple olfactory brains areas were listed (Supplementary Table S2). The percent of starter cells in the neurons infected by AAV-Dio-TVA-GFP in HMSc group higher than those in the other two group (18.52 ± 1.22% for HMSc; 13.20 ± 1.60% for MS/DBc; 15.04 ± 0.65% for NBMc; *F*_(2,21)_ = 9.855, *p* = 0.001, Figure 1D). Most of these starter cells were cholinergic (92.97% ± 1.75% for MS/DBc; 91.05% ± 0.77% for HMSc; 93.09% ± 1.60% for NBMc, all *p* values > 0.05, Figure 1E). To reduce the error caused by unequal numbers of starter cells, the percent of every brain area input was normalized by the total number of input neurons. The total percentage in the olfactory areas showed that the cholinergic neurons in HMSc received more olfactory inputs than those in MS/DBc and NBMc (34.37 ± 5.72% for HMSc; 9.59 ± 3.53% for MS/DBc; 20.50 ± 9.69% for NBMc; *F*_(2,6)_ = 4.986, *p* = 0.053, *post hoc* test *p* = 0.02, Figures 2A). A total of 13 olfactory regions were chosen to detail the olfactory inputs (Figures 2B,C). The 13 olfactory regions include OB, anterior olfactory nucleus (AON), PC, hippocampus (HIP), retrohippocampal region (RHP), olfactory tubercles (OT), taenia tecta (TT), dorsal peduncular area (DP), anterior amygdala area (AAA), piriform-amygdala area (PAA), central amygdala nucleus (CEA), basolateral amygdala nucleus (BLA) and cortical amygdala area (COA). The cholinergic neurons in HMSc received inputs from all olfactory regions, especially the PC (14.16 ± 3.41% for total and 41% for the olfactory) and HIP (5.62 ± 0.25% for total and 16% for the olfactory); those in MS/DBc received the highest inputs from HIP (5.44 ± 1.60% for the total and 57% for the olfactory); and those in NBMc primarily received amygdala nucleus such as CEA (13.10 ± 3.84% for the total and 71% for the olfactory; Figures 2B,C). Meanwhile, the three subpopulations of BFCNs showed diverse olfaction inputs (*r* = 0.466, *p* = 0.109 for MS/DBc vs. HMSc; *r* = 0.128, *p* = 0.677 for HMSc vs. NBMc;* r* = 0.029, *p* = 0.926, for MS/DBc vs. NBMc; Figures 2B,C). The results showed the different subpopulations of BFCNs receive diverse inputs from the olfactory system, forming distinct inputs patterns.

**Figure 3:**
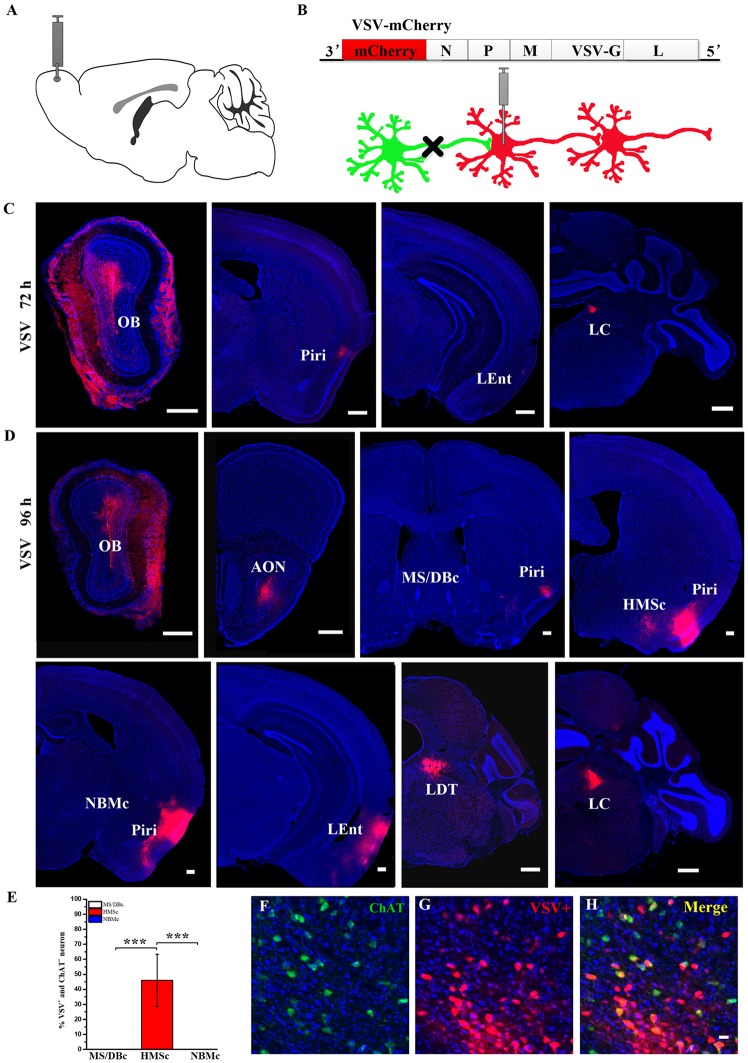
The outputs of the olfactory bulb (OB) to the BF. **(A)** Diagram of vesicular stomatitis virus (VSV) injection site. **(B)** Schematic of VSV anterograde transsynaptic labeling. **(C)** VSV infected olfactory relative brain regions by 72 h. **(D)** VSV infected olfactory relative brain regions by 96 h. Neurons (red) were infected by VSV. Scale Bar, 500 μm. **(E)** Quantitative analysis of the cholinergic neurons infected by VSV in different subsets of the BF. **(F–H)** Confirmation of cholinergic neurons infected by VSV in HMSc. ChAT+ neurons by anti-Choline Acetyltransferase (green), VSV+ neurons (Red), Merge (Yellow): ChAT+ neurons infected by VSV. Scale bar, 20 μm. *n* = 4. *P < 0.05, **P < 0.01 and ***P < 0.001.

Although the input networks of the BFCNs as a whole have been reported, the input networks of different subsets of BFCNs from the olfactory system and their comparisons have not been studied. Therefore, we would like to use an anterograde tracer, VSV, to partially confirm what we have found above (Figures 3A,B). The OB was chosen as the validation site because it receives all periphery olfactory inputs and projects only to HMSc (Figures 2B,C), not the other two subregions. To ensure the accuracy of VSV result, CTB diffusion range was carefully checked to confirm that their locations were restricted to OB (Supplementary Figure S2). Although the neurons infected by VSV in the injection site had morphological changes on account of VSV cytotoxic effect, VSV positive neurons including MC could be found in OB (Figures 3C,D, Supplementary Figure S2). The tracing results showed few neurons in several olfactory relative brain regions were VSV infected by 72 h, such as AON, PIR and locus coeruleus (LC; Figure 2C). By 96 h, more neurons were infected in multiple regions, including AOB, PIR, LEnt, HMSc, LC and lateral tegmental (LDT; Figure 3D). Most of them were olfaction system relative brain areas. Moreover, BF was infected by VSV, and the dsred-positive neurons were solely located in HMSc, not in MS/DBc and NBMc (Figures 3D,E). To confirm that some of the infected neurons are cholinergic, immunohistochemical staining for ChAT was performed. The results showed that approximately 50% of the VSV infected neurons was ChAT-positive (*F*_(2,21)_ = 39.099, *p* < 0.001; Figures 3E–H). Therefore, the anterograde tracing results were consistent with the AAV-RV retrograde tracing for the connections between the OB and the different BFCN subsets.

### BF Neurons in Different Subregions Were Activated Differentially in Go/No-Go Olfactory Discrimination Task

**Figure 4:**
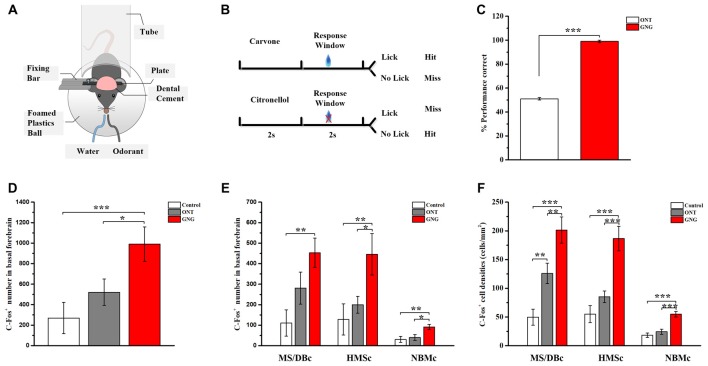
BF neurons were activated in go/no-go olfactory discrimination task. **(A,B)** Diagrams of olfactory go/no-go discrimination task. **(C)** Behavioral performance of two groups during test (day 6) following 5-day training in olfactory go/no-go discrimination task. **(D)** Total amount of the activated BF neurons among control (white), ONT (gray) and GNG group (red). The quantity of the activated BF neurons in GNG group was higher than those in control group and ONT group. **(E,F)** The total quantity and mean density of activated BF neurons in different subpopulations. The quantity and density of activated neurons in each subpopulation of BF in GNG group was higher than those in control group and ONT group. Data are showed as mean ± SEM. *P < 0.05, **P < 0.01 and ***P < 0.001; one-way ANOVA was used. *n* = 5.

Olfactory learning and memory involve complex neural circuits made up of neurons from numerous brain areas, including BF neurons. However, how these different subregions of BF differ in olfactory associated memory has not been studied. Activation of c-fos was used to map the activated neurons during the corresponding behavior. C57BL/6 mice were first trained to learn go/no-go olfactory discrimination task (Figures 4A,B). After 5-day training, the correct rate of the performance was attained at > 90%. The trained animals were tested on the 6th day, showing that the memory was retained (Figure 4C). Quantitative analysis of the activated BF neurons revealed significant differences among the three groups (*F*_(2,12)_ = 7.328, *p* = 0.008, Figure 4D). Similar results could be found in amygdala as basolateral amygdalar nucleus (BLA; 8.89 ± 1.00% for Control group, 132.78 ± 18.69% for ONT group, 236.95 ± 31.22% for GNG; *F*_(2,12)_ = 32.628, *p* < 0.001, Supplementary Figure S3). The c-fos immumostaining results showed that more neurons in GNG group were activated than the control and ONT groups in the BF as a whole, but there was no significant difference between the ONT and control groups in spite of a rising trend (990 ± 150.73 for GNG, 269.60 ± 136.67 for control, and 520.60 ± 115.52 for ONT groups). More detailed analysis revealed that in all the three different BF subpopulations, the total amount and mean density of c-fos immunopositive cells were obviously increased in GNG group than control or ONT groups (all *F*-values > 5.865, all *p*-values < 0.05, Figures 4E,F). The activated BF neurons of different subpopulations showed similar distributed patterns among the three experimental groups. The total amount and the average density of the activated neurons in SIc and MS/DBc are similar, both higher than NBMc in GNG group (total amount: 453 ± 71.70 for MS/DBc, 445.4 ± 101.10 for HMSc, 91.6 ± 12.99 for NBMc; average density: 201.34 ± 22.91% for MS/DBc, 186.56 ± 21.32% for HMSc, 54.83 ± 4.66% for NBMc). These results suggest the BF neurons are involved in olfactory associated memory, although not equally.

### BFCNs Were Activated Differentially in Go/No-Go Olfactory Discrimination Task

**Figure 5:**
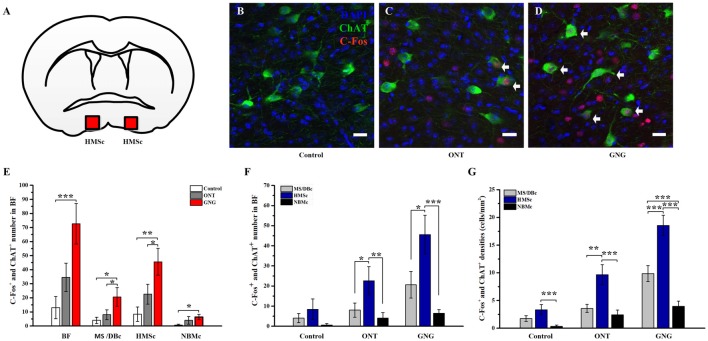
BFCNs were activated and showed a characteristic distribution pattern in go/no-go olfactory discrimination task. **(A)** The square in coronal atlas example indicated the site of HMSc where the cholinergic neurons were activated. **(B–D)** Representative coronal sections showing co-expressions of c-Fos+ (Red) and ChAT+ neurons (Green) in HMSc for the three paradigms. Scale bar, 20 μm. The arrows point to neurons co-expressing c-Fos and ChAT. **(E)** BFCNs were activated and showed similar activated patterns among the three groups in go/no-go olfactory discrimination task. In GNG group, the numbers of activated cholinergic neurons in the whole BF and in different subsets were higher than those in control group and ONT group. **(F–G)** The total quantity and mean density of the activated BFCNs were higher in HMSc than MS/DBc and NBMc among the three groups, and especially go/no-go group. Data are showed as mean ± SEM. *P < 0.05, **P < 0.01 and ***P < 0.001; one-way ANOVA was used. *n* = 5.

**Figure 6:**
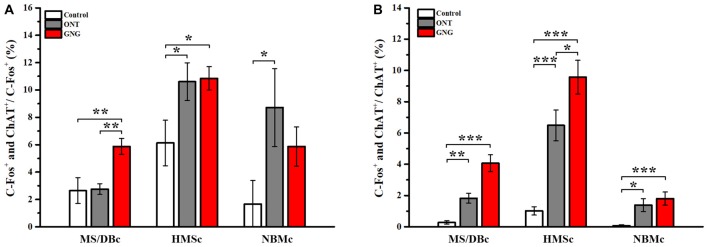
Proportion of the activated cholinergic neurons in total activated neurons and total cholinergic neurons in different subpopulations. **(A)** Proportion of the activated BFCNs over the total c-fos+ cells in different behavioral paradigms. In GNG group, the proportion of the activated cholinergic neurons in HMSc was much higher than the other two subpopulations. Moreover, higher percentages of the activated BFCNs were found in HMSc and NBMc in ONT group. **(B)** Proportion of the activated BFCNs over the total ChAT+ cells in different behavioral paradigms. Among the three sub-groups, the highest proportion of the activated cholinergic neurons was in HMSc GNG group. Data are showed as mean ± SEM. *P < 0.05, **P < 0.01 and ***P < 0.001; one-way ANOVA was used. *n* = 5.

Our tracing data have shown that the different subsets of BFCNs have different olfactory inputs and the c-fos mapping data demonstrate that these different regions are indeed activated unequally. To reveal the involvement of different subpopulations of BFCNs in these behavioral paradigms, double immunostainings for c-fos and ChAT were performed. Coronary SIc slices were exemplified and shown both c-fos and ChAT positive neurons among different paradigms (Figures 5A–D). Quantitative analysis of the doubly stained neurons showed that the activated BFCNs as a whole were significantly increased in GNG group than the other two groups (72.60 ± 12.89 for GNG, 13.0 ± 7.09 for control, and 34.60 ± 20.12 for ONT groups, respectively; *F*_(2,12)_ = 9.180, *p* = 0.004, Figure 5E, left panel). Further analysis revealed that the activated BFCNs in different subsets had similar patterns under different behavioral paradigms (Figure 5E, the three panels on the right). Generally, the BFCNs in all the three subregions were activated with qualitatively similar patterns in the three experimental paradigms: GNG > ONT > Control group. Quantitatively, both the density and the total number of the activated neurons in GNG group were the highest in each subpopulation among the three groups (HMSc:* F*_(2,12)_ = 7.891, *p* = 0.006; MS/DBc: *F*_(2,12)_ = 4.618, *p* = 0.033; NBMc: *F*_(2,12)_ = 2.889, *p* = 0.095), which is different from the patterns for all neurons that were activated (Figure 4E). Detailed comparison revealed that the patterns of the three experimental paradigms for the total quantity (Figure 5F) and mean density (Figure 5G) of the activated BFCNs were similar: Control < ONT < GNG, and NBMc < MS/DBc < HMSc (total quantity: *F*_(2,12)_ = 1.812, *p* = 0.205 for control group, *F*_(2,12)_ = 5.185, *p* = 0.024 for ONT, *F*_(2,12)_ = 10.667, *p* = 0.002 for GNG group, Figure 5F; all *F*-values > 5.815, all *p*-values < 0.005, Figure 5G). Since the areas of these three sub-regions are different, the density of the c-fos+ cells was analyzed. The mean density of the activated BFCNs was robustly different among these different regions, with the highest proportion in the HMSc GNG group (Figures 6A,B). In MS/DBc, the percentage of double labeled neurons over the total c-fos+ cells was higher in GNG group than those in control and ONT groups (5.87% ± 0.56% for GNG, 2.76% ± 0.38% for ONT, and 2.64% ± 0.91% for control, respectively; *F*_(2,42)_ = 7.788, *p* = 0.001; Figure 6A, left panel). In HMSc, the activated ChAT neurons in ONT and GNG group took similar percentages, much higher than the control group (10.84% ± 0.83% for GNG, 10.61% ± 1.34% for ONT, and 6.13% ± 1.62% for control, respectively; *F*_(2,57)_ = 4.139, *p* = 0.021; Figure 6A, middle panel). In NBMc, the proportion in ONT group was much higher than those in the control and GNG group (5.87% ± 1.39%for GNG, 8.72% ± 2.77% for ONT, and 1.67% ± 1.67% for control, respectively; *F*_(2,57)_ = 3.043, *p* = 0.056; Figure 6A, right panel). Among the three subregions, HMSc has the highest density of activated ChAT+ cells. Compared with the percentage over the total c-fos+ cells, the density of the activated ChAT neurons over the total ChAT+ cells, the trends were more regular. The density was GNG > ONT > Control for all subregions and similarly, that in the SIc had the highest activated ChAT density (Figure 6B). These results demonstrate that the BFCNs are activated differentially in go/no-go olfactory discrimination task, with these in HMSc involved the most.

## Discussion

In this study, the viral tracing results revealed that the different subpopulations of BFCNs in HMSc, NBMc and MS/DBc received diverse inputs from the olfactory system differentially, and the c-fos mapping results demonstrated that these different subpopulations of BFCNs were activated by an olfaction associated learning/memory task differentially. These results provided new structural and functional information for the relationship between the different subsets of BFCNs and the olfactory system.

### The BFCNs in HMSc May Have the Strongest Relation With the Olfaction System

Previous studies have demonstrated that BFCNs are important for olfactory memory (Roman et al., 1993; Ravel et al., 1994; Linster et al., 2001). Our tracing results revealed that 34% of the total inputs to the BFCNs in SIc were from the olfactory system, highest among the three subsets of cholinergic neurons. Of the 13 olfactory areas, the inputs from in the PC and HIP make up to 57% of the total (Figure 2C). This anatomical connection is quantitatively in agreement with the earlier studies (Do et al., 2016; Hu et al., 2016). PC is the primary olfactory cortex which plays crucial roles in the coding of odor quality, odor discrimination and olfactory associated memory (Roullet et al., 2005; Roland et al., 2017), while the HIP is a key brain region for episodic memory (Squire and Zola-Morgan, 1991; Ryan et al., 2010). Therefore, from the structural connections, we can deduce that the BFCNs in HMSc should play important roles in olfactory associated learning/memory. Indeed, our c-fos mapping results revealed that the BFCNs in HMSc were highly activated among the three subsets of BFCNs during odor associated go/no-go task (Figures 5E–G, 6B). Furthermore, although the total number and the density of the activated neurons in HMSc were equivalent to these in MS/DBc (Figures 4E,F), the number and density of the activated cholinergic neurons in HMSc were significantly higher during go/no-go task (Figures 5F,G, 6B), showing that the BFCNs in HMSc are selectively activated compared with the other two subregions.

### The BFCNs in NBMc and MS/DBc Might Be Strongly Involved in Olfaction Associated Memory and Emotion, Respectively

Our tracing results showed that 9.6% and 20.5% of the total inputs onto the BFCNs in MS/DBc and NBMc were from the olfactory system, respectively. Similar to the situation in HMSc, one area dominates the olfactory inputs to these BFCNs: for MS/DBc, HIP takes 57% of the total, while for NBMc, amygdala takes over 90% of the total (Figure 2C). Amygdala, part of the limbic system, plays crucial roles in a variety of functions, such as the generation and modulation of emotion, and the regulation of learning and memory (Hitchcock and Davis, 1986; Hitchcock et al., 1989; Kesner et al., 1989; Zola-Morgan et al., 1991). Therefore, based on the inputs, we can speculate that the BFCNs in MS/DBc are more involved in memory-related task, while the BFCNs in NBMc are more related to emotions. Indeed, the c-fos mapping results for the activated neurons in mice performed memory related task (odor associated go/no-go) demonstrated that the number of the activated BFCNs in MS/DBc doubled that in NBMc (Figure 5F), despite the total olfactory inputs to the BFCNs in MS/DBc halved that in NBMc (Figure 2A). Therefore, our experimental results on the structural connections of the BFCNs with the olfactory system provide a solid basis for their functional roles associated with olfaction.

### The Tracing Techniques and the Classification of the BFCN Subsets in This Study

It is hard to reveal the input networks of a given type of neurons using classical tracers, such as fluorescent dyes. In this study, the retrograde trans-monosynaptic AAV-RV tracing results showed the olfactory system projects differentially onto the different BFCN subpopulations (Figures 2A–C). The reliability of the results was enhanced by the anterograde tracing data (Figure 3). VSV tracing revealed that only HMSc receives inputs from the OB (Figure 3). This is exactly the results from the retrograde tracing from these three subregions (Figures 2B,C). Further, compared with the involvement of these BFCNs in odor associated learning/memory task as reflected by the number and density of the activated cholinergic neurons in these three subregions, the structural connection and the functional involvement agree with each other well. However, the labeling efficiency for a given type of neuron with AAV-RV system has not been characterized yet and whether these viruses have different efficiencies in infection, transportation, replication and transsynaptic spreading in the neurocircuits of different BFCNs are also unknown. More detailed characterization of the tracing systems and the exact quantitative information for these circuits require more systemic works in future.

Another shortcoming in this study is the lack of specific molecular markers for these different subtypes of BFCNs. The BFCNs are rather heterogeneous, based on their input and output networks, and brain functions involved. However, different subsets of BFCNs are located geologically close, and the shapes of these small subregions are irregular. Without specific markers to access each subtype of BFCNs (such as cre-line), it is almost impossible to map out the networks of a given subtype completely and specifically, by solely relying on injection site of the tracing virus. Therefore, the three subtypes of BFCNs are not classified purely according to the known subregions (Mesulam et al., 1983). Works seeking for specific markers are undergoing project so as to generate better tools for the relevant studies in future.

In summary, we mapped out the input networks from the olfactory system for three different subpopulations of BFCNs: each subset has a distinguished input pattern. Furthermore, each subtype of these BFCNs were activated differentially in an odor associated memory task. These results lay an anatomical foundation for understanding the potential mechanisms how the BFCNs are involved in various types of olfactory associated functions.

## Author Contributions

YZ and FX: conceptualization, writing and data analysis. SF, XZ, WJ, PW and FY: collected the data. SJ, XR and XH: guided viruses use.

## Conflict of Interest Statement

The authors declare that the research was conducted in the absence of any commercial or financial relationships that could be construed as a potential conflict of interest.

## Supplementary Material

FIGURE S1 Quantitative analysis of the AAV-RV whole-brain inputs for different subregions of BFCNs. **(A)** Quantitative analysis of whole-brain inputs to BFCNs in MS/DBc. **(B)** Quantitative analysis of whole-brain inputs to BFCNs in HMSc. **(C)** Quantitative analysis of whole-brain inputs to BFCNs in NBMc. The values in every brain area was normalized by the total number of input neurons. Less than 0.5% average input proportions were excluded.

FIGURE S2 The diffusion range of VSV near injection site. The representative coronal sections of OB injected VSV and CTB 488 were selected from anterior to posterior of OB. The left columns represent the diffusion range of VSV and CTB 488 in OB. The green neurons were combined with CTB 488. More CTB 488 positive neurons were found near the injection site. The red neurons were infected by VSV in OB (second column). CTB 488 diffusion rang near injection site in OB (third column). The last column represented the magnifying representative regions of neurons combined with CTB.

FIGURE S3 Amygdaloid neurons were activated in go/no-go olfactory discrimination task. **(A)** The representative coronal sections of the basolateral amygdalar nucleus (BLA) showing c-Fos+ (Red) and ChAT+ neurons (Green) in different behavioral paradigms. Scale bar, 100 μm. **(B)** Mean densities of the activated amygdala neurons among control (gray), ONT (blue) and GNG groups (black). The average densities of the activated amygdala neurons in GNG group was higher than those in control and ONT groups. **P* < 0.05, ***P* < 0.01 and ****P* < 0.001.

TABLE S1 Detailed information of virus used in the present article.

TABLE S2 The numbers of neurons of multiple olfactory brain areas inputs to BFCNs in different subpopulations.
