# Supplementary figures and images for: Different Subgroups of Cholinergic Neurons in the Basal Forebrain Are Distinctly Innervated by the Olfactory Regions and Activated Differentially in Olfactory Memory Retrieval
Source: Front Neural Circuits. 2018 Nov 13;12:99. doi: 10.3389/fncir.2018.00099 (PMC6243045; doi:10.3389/fncir.2018.00099)

A

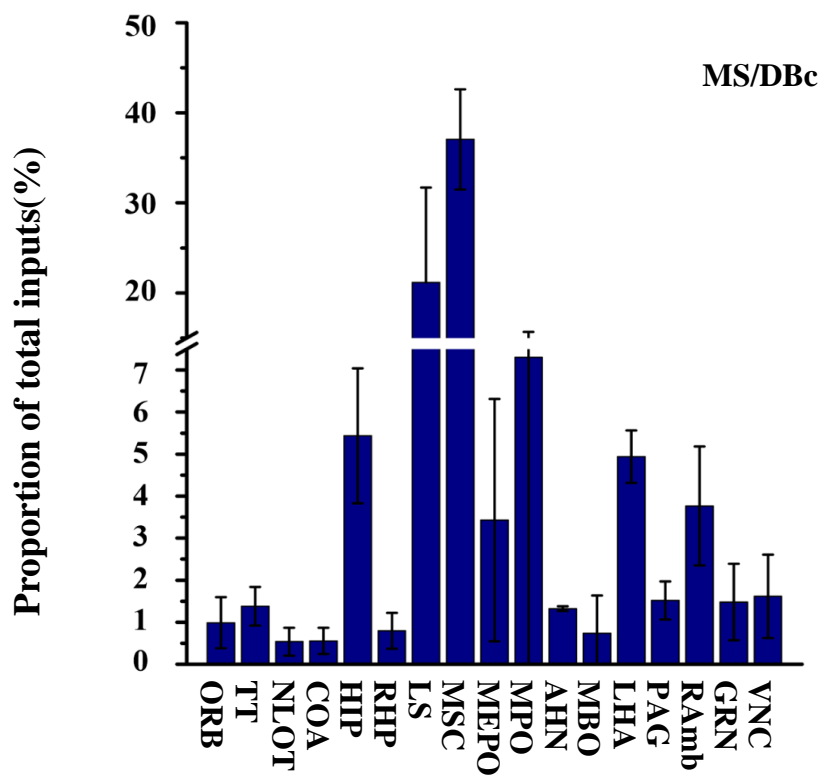

B

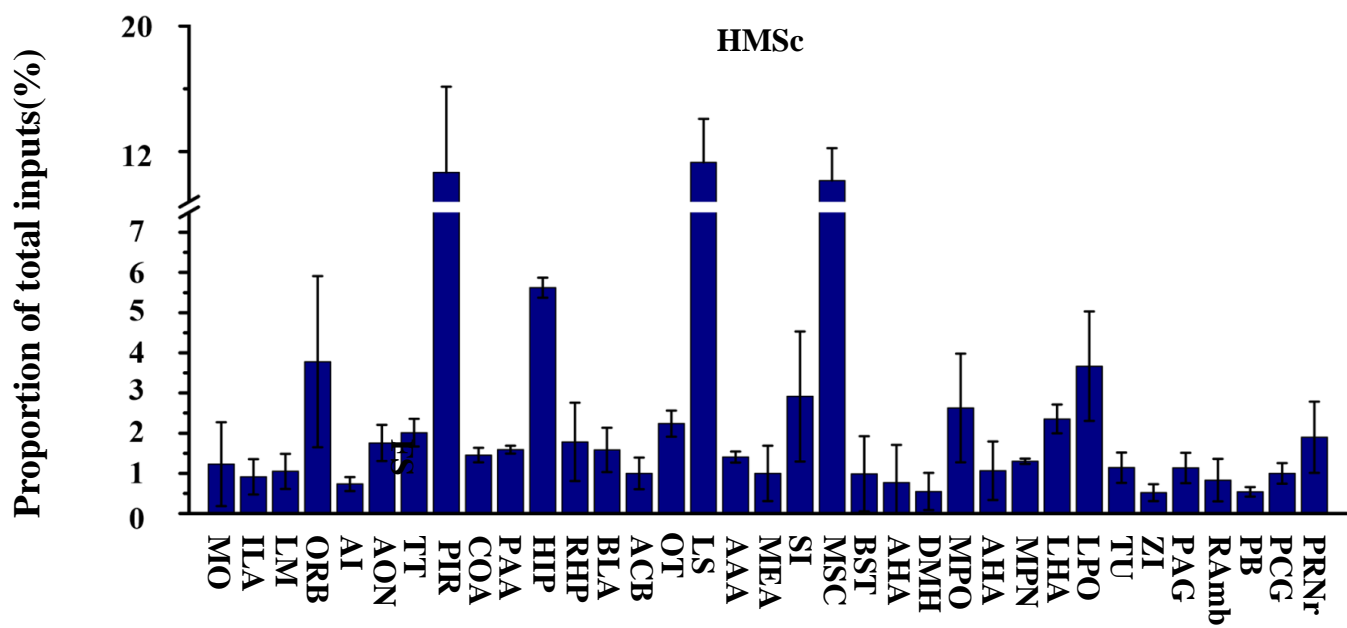

C

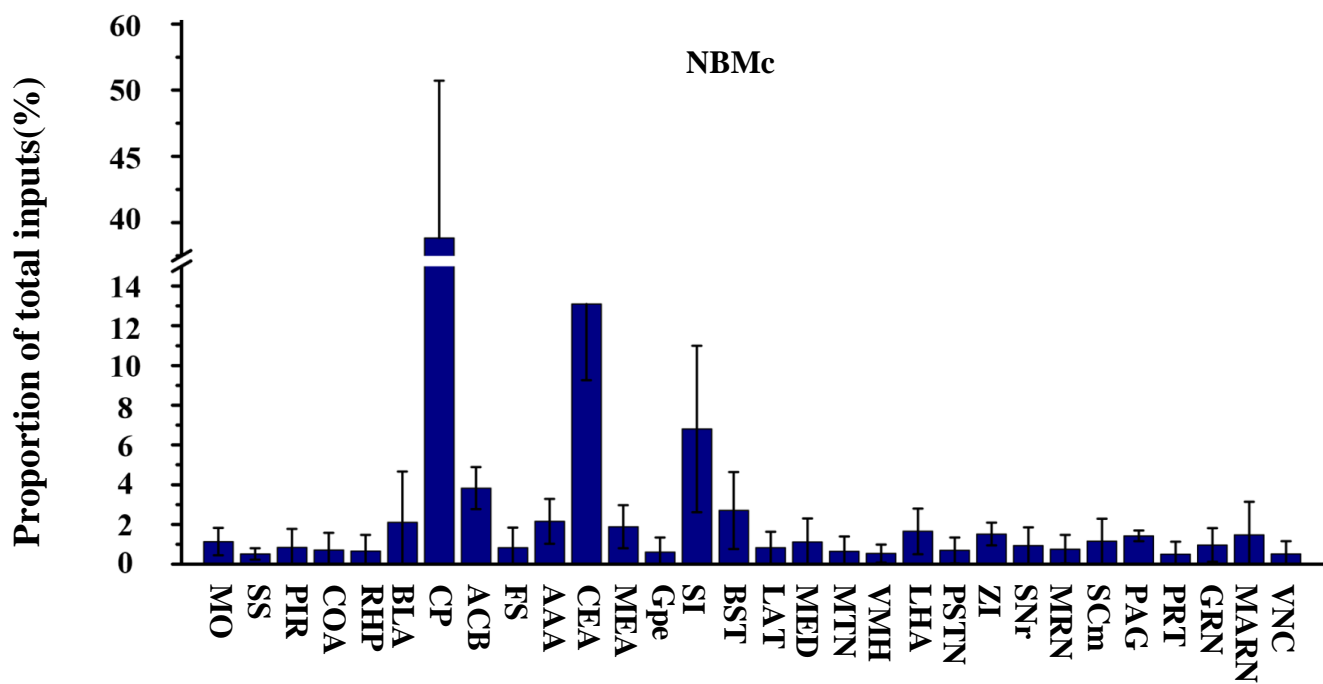

Supplement: FIGURE S1 — Quantitative analysis of the AAV-RV whole-brain inputs for different subregions of BFCNs. (A) Quantitative analysis of whole-brain inputs to BFCNs in MS/DBc. (B) Quantitative analysis of whole-brain inputs to BFCNs in HMSc. (C) Quantitative analysis of whole-brain inputs to BFCNs in NBMc. The values in every brain area was normalized by the total number of input neurons. Less than 0.5% average input proportions were excluded. [file Image_1.pdf]

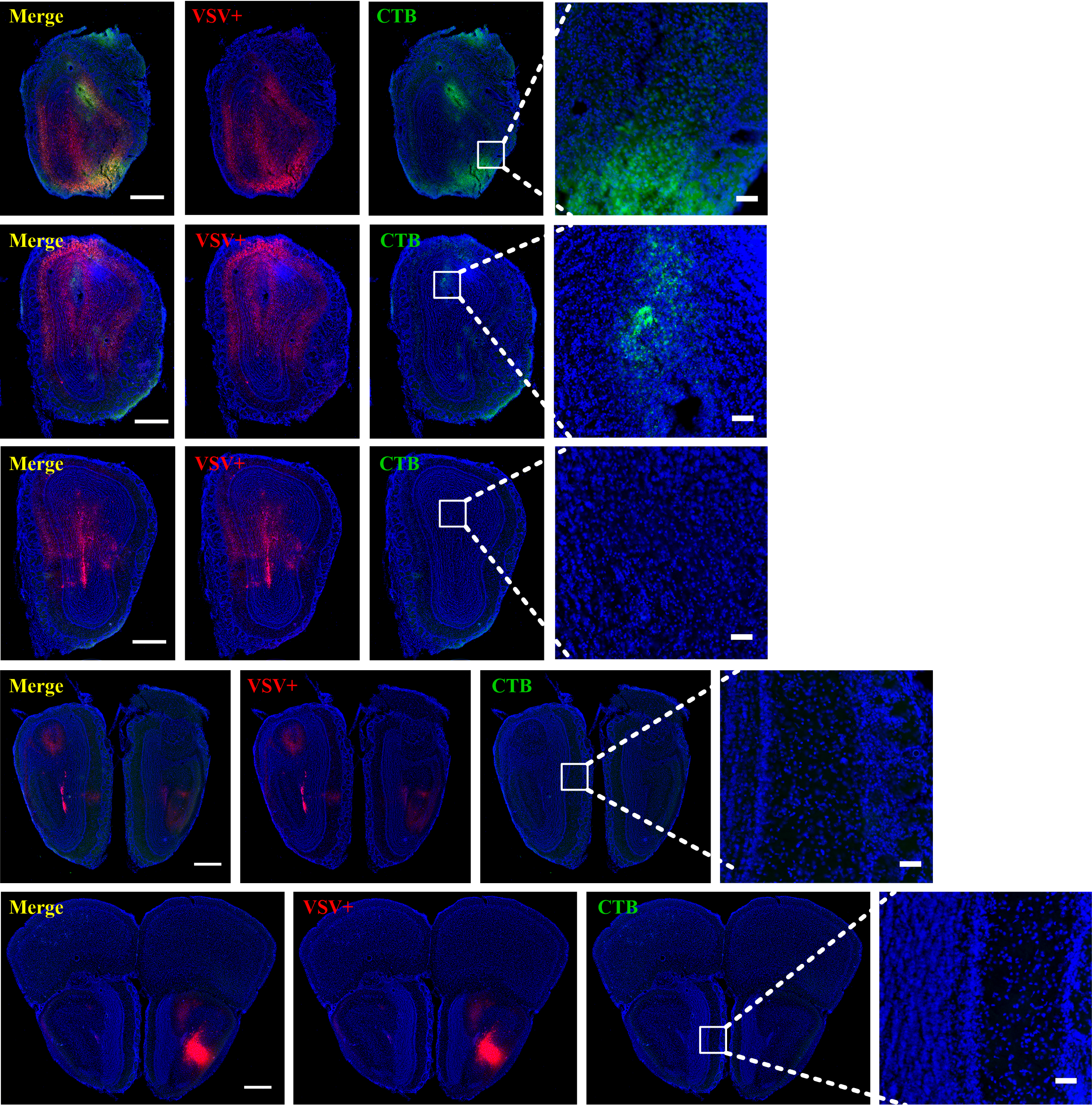

Supplement: FIGURE S2 — The diffusion range of VSV near injection site. The representative coronal sections of OB injected VSV and CTB 488 were selected from anterior to posterior of OB. The left columns represent the diffusion range of VSV and CTB 488 in OB. The green neurons were combined with CTB 488. More CTB 488 positive neurons were found near the injection site. The red neurons were infected by VSV in OB (second column). CTB 488 diffusion rang near injection site in OB (third column). The last column represented the magnifying representative regions of neurons combined with CTB. [file Image_2.TIF]

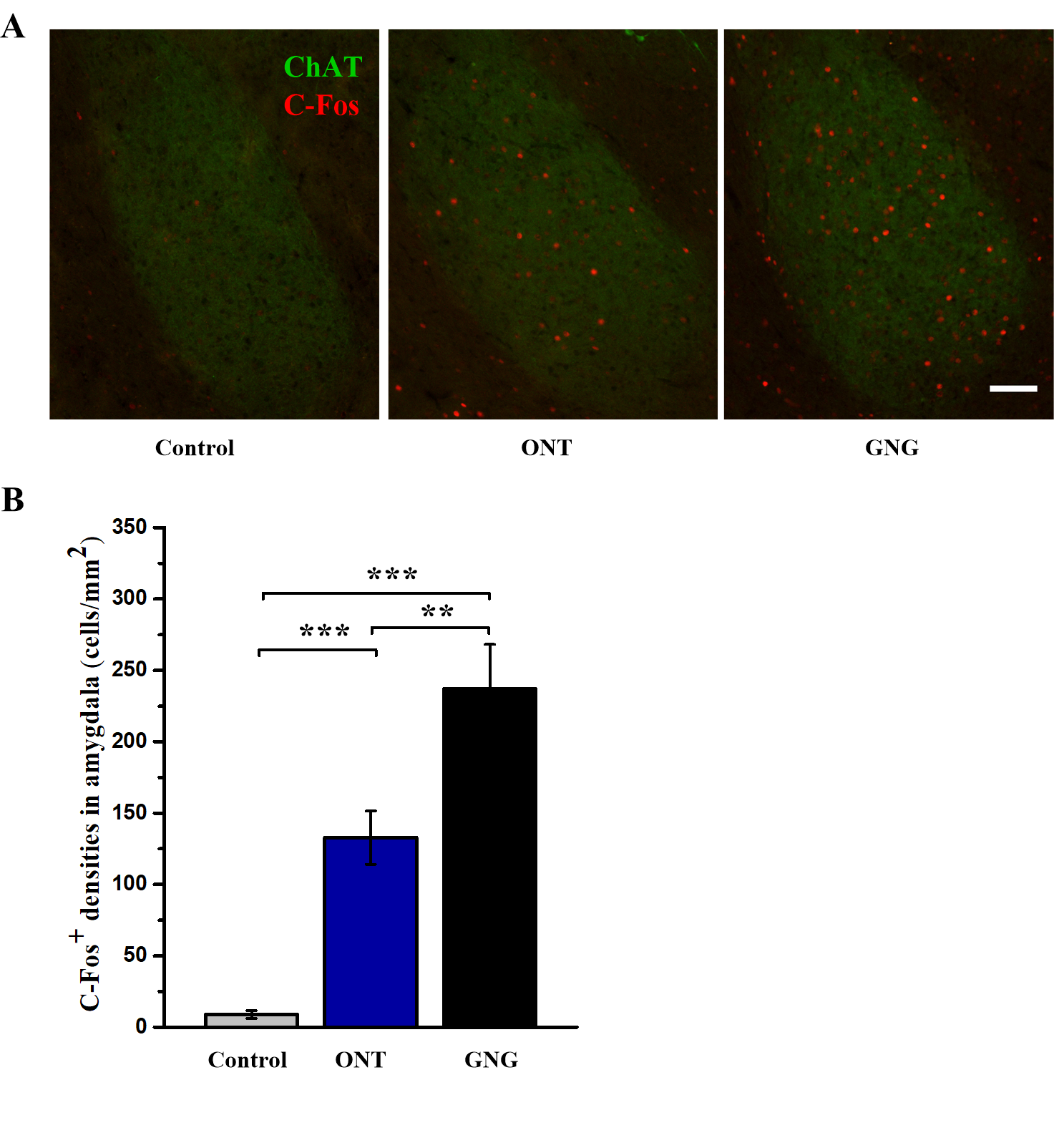

Supplement: FIGURE S3 — Amygdaloid neurons were activated in go/no-go olfactory discrimination task. (A) The representative coronal sections of the basolateral amygdalar nucleus (BLA) showing c-Fos+ (Red) and ChAT+ neurons (Green) in different behavioral paradigms. Scale bar, 100 μm. (B) Mean densities of the activated amygdala neurons among control (gray), ONT (blue) and GNG groups (black). The average densities of the activated amygdala neurons in GNG group was higher than those in control and ONT groups. *P < 0.05, **P < 0.01 and ***P < 0.001. [file Image_3.TIF]
