# Supplementary material for: Different Subgroups of Cholinergic Neurons in the Basal Forebrain Are Distinctly Innervated by the Olfactory Regions and Activated Differentially in Olfactory Memory Retrieval
Source: Front Neural Circuits. 2018 Nov 13;12:99. doi: 10.3389/fncir.2018.00099 (PMC6243045; doi:10.3389/fncir.2018.00099)
Supplement: TABLE S1 — Detailed information of virus used in the present article. [file Table_1.docx]

Supplementary Table 1. Detailed information of virus used in the present paper.

| Name | Titer (genomix copies /ml) |
| --- | --- |
| AAV-CAG-Dio-TVA-GFP | 5×10^12^ |
| AAV-CAG-Dio-RVG | 6.8×10^12^ |
| RV-Enva-dsRed | 5.0×10^8^ |
| VSV-mCherry | 5.0×10^7^ |

Supplementary Table 2 Comparison of olfactory system afferent to the subpopulation of BFCNs.

| Brain areas | Abbreviations | Comparison of olfactory inputs | | |
| --- | --- | --- | --- | --- |
|  |  | MS/vDB: SI | MS/vDB: NBM | SI:NBM |
| Anterior amygdalar area | AAA | *P*>0.05 | *P<*0.05 | *P*>0.05 |
| Anterior olfactory nucleus | AON | *P*=0.002 | *P*>0.05 | *P*=0.002 |
| Basolateral amygdalar area | BLA | *P*>0.05 | *P*>0.05 | *P*>0.05 |
| Central amygdalar nucleus | CEA | *P*>0.05 | *P*=0.002 | *P*=0.002 |
| Cortical amygdalar area | COA | *P*>0.05 | *P*>0.05 | *P*>0.05 |
| Dorsal peduncular area | DP | *P*>0.05 | *P*>0.05 | *P*>0.05 |
| Hippocampus | HIP | *P*>0.05 | *P*=0.003 | *P*=0.002 |
| Olfactory bulb | OB | *P*=0.002 | *P*>0.05 | *P*=0.002 |
| Olfactory tubercle | OT | *P*<0.001 | *P*>0.05 | *P*<0.001 |
| Piriform-amygdalar area | PAA | *P*<0.001 | *P*>0.05 | *P*<0.001 |
| Piriform area | PIR | *P*=0.001 | *P*>0.05 | *P*=0.001 |
| Retrohippocampal area | RHP | *P*>0.05 | *P*>0.05 | *P*>0.05 |
| Taenia tecta | TT | *P*>0.05 | *P*>0.01 | *P*=0.002 |

Note: **P*<0.05, ***P*<0.01 and ****P*<0.001; one-way ANOVA was used.
