# Supplementary material for: Different Subgroups of Cholinergic Neurons in the Basal Forebrain Are Distinctly Innervated by the Olfactory Regions and Activated Differentially in Olfactory Memory Retrieval
Source: Front Neural Circuits. 2018 Nov 13;12:99. doi: 10.3389/fncir.2018.00099 (PMC6243045; doi:10.3389/fncir.2018.00099)
Supplement: TABLE S2 — The numbers of neurons of multiple olfactory brain areas inputs to BFCNs in different subpopulations. [file Table_2.DOCX]

Supplementary Table 2. The numbers of neurons of multiple olfactory brain areas inputs to BFCNs in different subpopulations.

| **Brains areas** | **MS/DBc**  **(Mean ± se)** | **HMSc**  **(Mean ± se)** | **NBMc**  **(Mean ± se)** |
| --- | --- | --- | --- |
| **OB** | 0 | 4.67 ± 0.33 | 0 |
| **DP** | 6.33 ± 1.67 | 16 ± 16 | 0 |
| **AON** | 2.67 ± 1.33 | 43 ± 1.52 | 1.33 ± 0.88 |
| **TT** | 21.67 ± 4.33 | 56 ± 18.6 | 0.66 ± 0.66 |
| **PIR** | 6.67 ± 3.33 | 399.33 ± 153.83 | 14.33 ± 11.92 |
| **HIP** | 86 ± 14 | 147.67 ± 24.29 | 3 ± 2.51 |
| **OT** | 0.67 ± 0.67 | 60.67 ± 17.18 | 7 ± 2.65 |
| **RHP** | 12 ± 5 | 55 ± 33.56 | 10.66 ± 10.66 |
| **BLA** | 0.67 ± 0.33 | 60.67 ± 17.19 | 7 ± 2.65 |
| **COA** | 8.33 ± 3.67 | 40 ± 11.79 | 11.67 ± 11.17 |
| **PAA** | 0.67 ± 0.33 | 43 ± 10.41 | 4 ± 3.05 |
| **AAA** | 0 | 38.33 ± 10.48 | 36 ± 13.33 |
| **CEA** | 3 ± 3 | 4 ± 2 | 283.33 ± 86.33 |
| **Olfactory Input** | 149.67 ± 35.33 | 952.33 ± 311.63 | 406.33 ± 174.97 |
| **Whole brain input** | 1673 ± 188.76 | 2650.67 ± 505.75 | 2172.33 ± 547.47 |
